# Establishment and Characterization of a Functionally Competent Type 2 Conventional Dendritic Cell Line

**DOI:** 10.3389/fimmu.2018.01912

**Published:** 2018-08-24

**Authors:** Matteo Pigni, Devika Ashok, Mathias Stevanin, Hans Acha-Orbea

**Affiliations:** Department of Biochemistry CIIL, University of Lausanne, Épalinges, Switzerland

**Keywords:** dendritic cell, cell line, conventional DC subset, cDC1, cDC2, cell culture, spleen, mouse

## Abstract

Dendritic cells (DCs) are the most potent antigen presenting cells and possess an incomparable ability to activate and instruct T cells, which makes them one of the cornerstones in the regulation of the cross-talk between innate and adaptive immunity. Therefore, a deep understanding of DC biology lays the foundations to describe and to harness the mechanisms that regulate the development of the adaptive response, with clear implications in a vast array of fields such as the study of autoimmune diseases and the development of new vaccines. However, the great difficulty to obtain large quantities of viable non-activated DCs for experimentation have considerably hindered the progress of DC research. Several strategies have been proposed to overcome these limitations by promoting an increase of DC abundance *in vivo*, by inducing DC development from DC progenitors *in vitro* and by generating stable DC lines. In the past years, we have described a method to derive immortalized stable DC lines, named MutuDCs, from the spleens of Mushi1 mice, a transgenic mouse strain that express the simian virus 40 Large T-oncogene in the DCs. The comparison of these DC lines with the vast variety of DC subsets described *in vivo* has shown that all the MutuDC lines that we have generated so far have phenotypic and functional features of type 1 conventional DCs (cDC1s). With the purpose of deriving DC lines with characteristics of type 2 conventional DCs (cDC2s), we bred a new Batf3^−/−^ Mushi1 murine line in which the development of the cDC1 subset is severely defective. The new MutuDC line that we generated from Batf3^−/−^ Mushi1 mice was phenotypically and functionally characterized in this work. Our results demonstrated that all the tested characteristics of this new cell line, including the expression of subset-determining transcription factors, the profile of cytokine production and the ability to present antigens, are comparable with the features of splenic CD4^−^ cDC2s. Therefore, we concluded that our new cell line, that we named CD4^−^ MutuDC2 line, represents a valuable model for the CD4^−^ cDC2 subset.

## Introduction

Dendritic cells (DCs) are a heterogeneous lineage of innate immune cells with a unique and essential role in the initiation and orchestration of the adaptive immune response (1). Upon encounter with pathogens, DCs become activated and undergo a series of functional modifications that include induction of cytokine and chemokine production, regulation of surface marker expression and increase of antigen presentation efficiency (2).

Pathogen sensing by DCs is achieved by means of a broad group of surface receptors called pattern recognition receptors (PRRs). PRRs recognize specific molecular motifs, collectively known as microbe associated molecular patterns (MAMPs), which are conserved among pathogenic and non-pathogenic microorganisms (3, 4). One of the most studied groups of PRRs is the toll like receptor (TLR) family which in mouse comprises twelve members, namely TLR1-9 and TLR11-13 (4). To carry out their function, TLRs form binary protein complexes that can be either homodimeric or heterodimeric, as in the case of TLR1/2 and TLR2/6, (5, 6), and that recognize MAMPs with distinct ligand specificity. The recognition of a MAMP by its specific TLR dimer triggers a signaling cascade that culminates in the activation of DCs with consequent increase of surface markers, like MHC-II and co-stimulatory molecules, and regulation of several effector genes, including pro-inflammatory cytokines and chemokines (7, 8).

DCs constantly endocytose self and foreign antigens, but it is especially following activation and maturation that they become highly efficient at forming peptide-MHC complexes for antigen presentation to T cells (9). Endogenous self-antigens or intracellular pathogen-derived antigens are presented to CD8^+^ T cells through direct MHC-I presentation. Instead, exogenous antigens, endocytosed from the extracellular environment, can be presented either to CD4^+^ T cells, by means of peptide-MHC-II complexes, or to CD8^+^ T cells via the alternative MHC-I pathway known as cross-presentation (9–11). Together with antigen presentation through peptide-MHC complexes, the upregulation of co-stimulatory molecules and the induction of cytokine production that follow DC activation provide the three canonical signals required for T cell activation (2).

As previously mentioned, DCs are very heterogeneous and can be classified into several distinct subsets. At the steady state, DCs are roughly divided into non-lymphoid tissue DCs, which during inflammation circulate loaded with antigens from the peripheral tissues to the draining lymph nodes through the lymphatics (12, 13), and lymphoid tissue-resident DCs, which differentiate and dwell in the lymphoid organs (14, 15). Additionally, under inflammatory conditions, blood circulating monocytes can differentiate into a subset of DCs known as monocyte-derived/inflammatory DCs (moDCs) (12, 16–20). The steady-state DC population is composed of two main subsets: plasmacytoid DCs (pDCs), which are known to be major producers of IFNα during antiviral response (21, 22), and conventional DCs (cDCs) (23). CDCs are subdivided into type 1 cDCs (cDC1s) and type 2 cDCs (cDC2s) on the basis of their ontogeny (24). In particular, the cDC1 subset includes all the cDCs whose development depends on the basic leucine zipper ATF-like transcription factor 3 (Batf3) (25, 26) and on the interferon regulatory factor 8 (IRF8) (25, 27, 28), while the cDC2 subset comprises all the cDCs that are independent of these transcription factors and that, by contrast, develop in an interferon regulatory factor 4 (IRF4)-dependent manner (29–31). Notably, this ontogeny-based classification system can be extended to cDCs across separate organs and species (32), overcoming the confusion generated by the phenotypic variability of analogous cDC subsets in different tissues. The different DC subsets express a wide variety of subset- and tissue-specific surface markers (1, 32) among which CD8α, CD11b, and CD4 have been traditionally used in mouse to discriminate the splenic resident cDC1s (spl-cDC1s) and cDC2s (spl-cDC2s) (33).

This varied assortment of DC subsets reflects a diversified array of functional specificities in terms of pathogen sensing, cytokine production and antigen presentation (34, 35). For instance, cDC1s are characterized by high levels of TLR3 and TLR9 but display selective lack of TLR7, while cDC2s have a wider TLR profile but show limited or absent TLR3 expression (36). Upon activation, cDC1s produce considerable quantities of IL-12, while cDC2s are known to be poor producers of this cytokine (33, 37, 38). Additionally, cDC1s are specialized in MHC-I-mediated cross-presentation of extracellular antigens, and hence they are more addressed toward cytotoxic T lymphocyte priming, while cDC2s are mainly oriented to MHC-II-mediated presentation and helper T cell activation (39–41).

In the past years, our group has developed a new method to generate immortalized DC lines from the spleen of a murine model of multisystem histiocytosis named Mushi1 (multisystem histiocytosis line 1) (42). Mushi1 mice carry a transgenic construct that contains the simian virus 40 Large T-oncogene (SV40LgT) and an IRES-linked EGFP reporter under the control of the 5.7 kb CD11c proximal promoter that restricts the transgene expression almost exclusively to DCs (42, 43). Between 3 and 5 months of age, Mushi1 mice develop splenic tumors caused by tumorigenic transformation of spl-cDC1s (42). From the tumors, stable cell lines named MutuDCs (murine tumor DCs) can be derived (44). These cells can be easily cultured through standard procedures without additional growth factors and are stable in long term culture for a minimum of 40 passages. Meticulous analysis of MutuDCs has clearly demonstrated that they share with spl-cDC1s all the main phenotypic and functional characteristics including the cross-presentation ability, and hence we refer to them as MutuDC1s (44).

In this work, we describe the derivation of the new CD4^−^ MutuDC2 line from splenic tumors of Batf3^−/−^ Mushi1 mice and we illustrate how the selective absence of cDC1s in this genetic background (26) allowed to obtain a cell line whose features are different from spl-cDC1s. We also show and discuss the characterization of these cells demonstrating that their phenotype and function are consistent with their belonging to the CD4^−^ spl-cDC2 subset.

## Materials and methods

### Mice

C57BL/6JOlaHsd mice were purchased from Harlan Laboratories. Mushi1 (42), OT-I, and OT-II transgenic mice were maintained and bred in our own facility. Batf3^−/−^ mice in a C57BL/6J background (26) were provided by Prof. Kenneth M. Murphy (Washington University School of Medicine in St. Louis, St. Louis, MO). All the animals were housed and bred under specific pathogen free conditions and used at an age of at least 8 weeks. Genomic DNA from pups of Batf3^−/−^ x Mushi1 litters was extracted by incubation of ear samples at 95°C in 600 μL of 50 mM NaOH for 30 min followed by addition of 50 μL of 1 M Tris-HCl pH 8. The genomic DNA was used for genotypic screening by PCR using the following primers: pCD11c (5′-GGCAGCTGTCTCCAAGTTGCTCAG-3′) and RβGR1 (5′-GGGTCCATGGTGATACAAGGG-3′) (42). All animal experiments were performed after approval by the cantonal veterinary office (Service de la consommation et des affaires vétérinaires, Département du territoire et de l'environnement, Permission no. VD2490.1).

### Culture conditions and generation of MutuDC lines

Cells were kept in culture at 37°C in a humidified incubator with 5% CO_2_. Complete culture medium was composed as follows: IMDM+GlutaMAX™ Supplement (31980, GIBCO), 10 mM HEPES (15630, GIBCO), 0.075% NaHCO_3_ (from 7.5% NaHCO_3_ stock solution, 25080, GIBCO), 50 μM β-mercaptoethanol (31350, GIBCO), 8% heat inactivated FCS (tested for toxicity toward DC cultures), 50 U/mL penicillin, 50 μg/mL streptomycin (15070, GIBCO). Cells were harvested by treatment with a non-enzymatic cell dissociation buffer (5 mM EDTA, 20 mM HEPES in PBS). The derivation of the new CD4^−^ MutuDC2s was carried out as previously described for other MutuDCs (44, 45). Spleens from diseased Batf3^−/−^ Mushi1 were cut with a scalpel and filtered through a 40 μm cell strainer to obtain single cell suspensions. The splenocytes were seeded in serial two-fold dilution in a 24-well plate at a starting density ≥10^7^ cells/well. After 8–16 h, non-adherent cells were removed by washing the wells. The adherent cells were maintained in 24-well plates for 5–10 passages during which the wells were frequently washed to remove non-adherent/dead cells or debris, and culture medium was changed periodically. In the earliest passages the cells were always kept at high density and split at a maximum 1:2 dilution. When the cells became able to tolerate 1:6 splitting, the cultures were progressively expanded. The MutuDCs chosen for the characterization described in this work have been numbered 20956A (clone A from mouse HAO-20956).

### Light microscopy

Pictures of cells were obtained by photographing the cultures directly with the EVOS™ FL Color Imaging System (AMEFC4300, ThermoFisher SCIENTIFIC).

### Splenocyte isolation, staining, and antibodies for flow cytometry

Spleens were cut into small pieces with a scalpel and incubated for 20 min at 25°C in a freshly prepared collagenase D/DNase I solution composed as follows: RPMI 1640+GlutaMAX™ Supplement (61870, GIBCO), 2% FCS, 1 mg/mL collagenase D (11088866001, ROCHE), 40 μg/mL DNase I (10104159001, ROCHE). After the digestion, the spleens were filtered through a 40 μm cell strainer to obtain single cell suspensions. All the washing steps and acquisitions were carried out in FACS buffer (3% Fetal Bovine Serum, 5 mM EDTA in PBS). For the staining, the cells were incubated for 30 min on ice with a staining mix composed of the appropriate antibody combinations diluted in a 1:2 solution of FACS buffer and supernatant from hybridoma 2.4G2. The cells were analyzed immediately after staining or fixed in 1% paraformaldehyde at room temperature for 10 min, stored at 4°C and analyzed within 3 days after the staining. For intracellular staining the eBioscience™ Foxp3 / Transcription Factor Staining Buffer Set (00-5523-00, ThermoFisher SCIENTIFIC) was used according to manufacturer's instructions. Flow cytometric data were acquired with BD LSR-II or BD LSRFortessa cytometers (BD Biosciences) and analyzed with FlowJo (version 10.0.8r1, Tree Star, Inc.). The fluorochrome-conjugated monoclonal antibodies that were used were specific for: B220 (CD45R) (clone RA3-6B2, Alexa Fluor 700, eFluor 450, PE, PE-Cy7, eBioscience), CD4 (clone RM4-5, APC, Pacific Blue, PE-Cy7, BioLegend, or APC, eFluor 450, PE-Cy7, PerCP-Cy5.5, eBioscience), CD8α (clone 53-6.7, PE-Cy7, BD Biosciences, or APC, APC-eFluor 780, eFluor 450, PE-Cy7, PerCP-Cy5.5, eBioscience), CD11b (clone M1/70, APC, PE, BioLegend, or APC, APC-eFluor 780, eBioscience), CD11c (clone N418, APC, PacificBlue, PE-Cy7, BioLegend, or eFluor 450, PE, PE-Cy7 eBioscience), CD24 (clone M1/69, APC, BD Biosciences, or eFluor 450, eBioscience), CD40 (clone 1C10, APC, PE, eBioscience), CD80 (clone 16-10A1, Brilliant Violet 421, BioLegend, or APC, eBioscience), CD86 (clone GL1, Alexa Fluor 700, BioLegend, or APC, eBioscience), CD64 (FcγRI) (clone X54-5/7.1, PE, BioLegend), CD172a (clone P84, APC, PE, BD Biosciences), CD205 (clone NLDC-145, APC, BioLegend, or clone 205yekta, PerCP-eFluor 710, eBioscience), CD206 (MMR) (clone C068C2, PE, BioLegend), CLEC9A (CD370) (clone 7H11, APC, PE, BioLegend, or clone 42D2, PE, eBioscience), F4/80 (clone BM8, APC, BioLegend, or eFluor 450, eBioscience), FcεRIα (clone MAR-1, PE, BioLegend), FLT3 (CD135) (clone A2F10, PE, eBioscience), Gr-1 (clone RB6-8C5, APC, BioLegend, or PE, eBioscience), IFNγ (clone XMG1.2, PE, BD Biosciences, or PE, BioLegend, or PE, PE-Cy7, eBioscience), IRF4 (clone 3E4, PE-Cy7, eBioscience), IRF8 (clone V3GYWCH, APC, eBioscience), MHC-II (clone M5/114.15.2, PerCP, BioLegend, or Alexa Fluor 700, PE, eBioscience), PDCA-1 (CD317) (clone eBio129c, PE, eBioscience), TLR5 (clone ACT5, Alexa Fluor 647, BioLegend).

### TLR stimulation and cytokine detection

CD4^−^ MutuDC2s and MutuDC1s were seeded at a density of 2.5 × 10^5^ cells/cm^2^ in 96-well or 48-well plates and incubated for 24 h with 450 μL/cm^2^ of the following TLR agonists diluted in complete medium: Pam3CSK4 (150 ng/mL, tlrl-pms, InvivoGen), poly(I:C) (8.5 μg/mL, tlrl-pic, InvivoGen), LPS from *E. coli* (100 ng/mL, tlrl-peklps, InvivoGen), ultrapure flagellin from *B. subtilis* (100 ng/mL, tlrl-pbsfla, InvivoGen), FSL-1 (100 ng/mL, tlrl-fsl, InvivoGen), Gardiquimod™ (1 μg/mL, tlrl-gdqs, InvivoGen), CpG ODN 1826 (1 μM, TriLink BIOTECHNOLOGIES). In all the experiments each condition was plated in technical triplicate. The supernatants were analyzed by ELISA for the presence of IL-6, IL-10, IL-12/IL-23 p40, IL-12p70, and MCP-1(CCL2) using the following kits according to manufacturer's instructions: Mouse IL-6 ELISA Set (555240, BD Biosciences) or Mouse IL-6 ELISA Ready-SET-Go! (88-7064, eBioscience), Mouse IL-10 ELISA Set (555252, BD Biosciences) or Mouse IL-10 (Interleukin-10) ELISA Ready-SET-Go! (88-7104, eBioscience), Mouse IL-12 (p40) ELISA Set (555165, BD Biosciences), Mouse IL-12 (p70) ELISA Set (555256, BD Biosciences), Mouse CCL2 (MCP-1) ELISA Ready-SET-Go! (88-7391, eBioscience).

### RNA extraction, cDNA synthesis, and RT-qPCR

Total RNA from CD4^−^ MutuDC2s and MutuDC1s was extracted with the RNeasy Plus Mini Kit (74134, QIAGEN) according to manufacturer's instructions and stored in RNA secure (AM7005, Thermo Fisher SCIENTIFIC). The synthesis of cDNA was carried out using random nonamers and the M-MLV reverse transcriptase kit (M1701, Promega) or the SuperScript™ Reverse Transcriptase kit (18064014, Thermo Fisher SCIENTIFIC) according to manufacturer's instructions, with the addition of RiboLock RNase Inhibitor (EO0381, Thermo Fisher SCIENTIFIC). DNA/RNA hybrids were removed with RNase H (70054Y, Thermo Fisher SCIENTIFIC). cDNAs were purified using the QIAquick PCR Purification Kit (28104, QIAGEN). RNA and cDNA yields were quantified by Nanodrop spectrophotometry (Thermo Fisher SCIENTIFIC). RT-qPCR was carried out using KAPA SYBR® FAST qPCR kit for LightCycler®480 (KK4611, SIGMA-ALDRICH) on a LightCycler®480 (384-well plate, 5 μL reaction) from Roche Diagnostics. The following primers were used at the final concentration of 500 nM: TLR3 FW (5′-GCGTTGCGAAGTGAAGAA-3′), TLR3 REV (5′-TCGAGCTGGGTGAGATTT-3′), TLR5 FW (5′-CCTCATCTCACTGCATACC-3′), TLR5 REV (5′-TATTACCAACACGGGGCT-3′), ACTB FW (5′-CTGAACCCTAAGGCCAACCGTG-3′), ACTB REV (5′-GGCATACAGGGACAGCACAGCC-3′). Every sample was analyzed in technical triplicates.

### T cell activation assays

Ovalbumin-specific CD8^+^ and CD4^+^ T cells were isolated from spleens and lymph nodes (brachial, inguinal and mesenteric) of OT-I and OT-II mice, respectively, and purified using the following MACS or EasySep™ kits: CD4^+^ T Cell Isolation Kit, mouse (130-104-454, Miltenyi Biotec), CD8a^+^ T Cell Isolation Kit, mouse (130-104-07, Miltenyi Biotec), EasySep™ Mouse CD4+ T Cell Isolation Kit (19852, STEMCELL™ TECHNOLOGIES), EasySep™ Mouse CD8+ T Cell Isolation Kit (19853, STEMCELL™ TECHNOLOGIES). The T cell isolation kits were used following manufacturer's protocols except for the buffers that were prepared as follows: MACS buffer (0.5% FCS, 2 mM EDTA in PBS), EasySep buffer (2% FCS, 1 mM EDTA in PBS). A fraction of the purified T cells was stained with fluorochrome-conjugated monoclonal antibodies specific for TCR β chain (clone H57-597, Brilliant Violet 510, BioLegend) and for either CD4 (clone RM4-5, APC, BioLegend or eBioscience) or CD8α (clone 53-6.7, APC, eBioscience) and analyzed by flow cytometry to assess T cell purity. The purified T cells were stained with the cell proliferation dye eFluor™ 670 (65-0840, eBioscience) or eFluor™ 450 (65-0842, eBioscience). CD4^−^ MutuDC2s and MutuDC1s were plated in 96-well round bottom plates at a density of 10^4^ cells/well and incubated for 6–8 h with the ovalbumin-derived peptides OVA_332−339_ (Protein and Peptide Chemistry Facility, UNIL) or OVA_257−264_ (Protein and Peptide Chemistry Facility, UNIL) or with the full-length ovalbumin (vac-pova, InvivoGen) in the presence of CpG ODN 1826 (1 μM, TriLink BIOTECHNOLOGIES). At the end of the incubation, the supernatants were removed and the wells were washed gently with fresh complete medium. The proliferation dye-labeled T cells were plated with the MutuDCs at a density of 10^5^ cells/well. After 3 days (CD8^+^ T cells) or 4 days (CD4^+^ T cells) of co-culture, the supernatant was removed from each well and the cells were restimulated for 6 h with phorbol 12-myristate 13-acetate (PMA) (10 ng/mL, P8139, SIGMA-ALDRICH), ionomycin (500 ng/mL, I0634, SIGMA-ALDRICH) in the presence of brefeldin A (00-4506-51, eBioscience). The T cells were stained with fluorochrome-conjugated monoclonal antibodies and analyzed by flow cytometry for proliferation and IFNγ production.

### Statistical analyses

All the statistical analyses were carried out using GraphPad Prism 7.04.

## Results

### Generation of batf3^−/−^ mushi1 mice and derivation of CD4^−^ MutuDC2s

**Figure 1:**
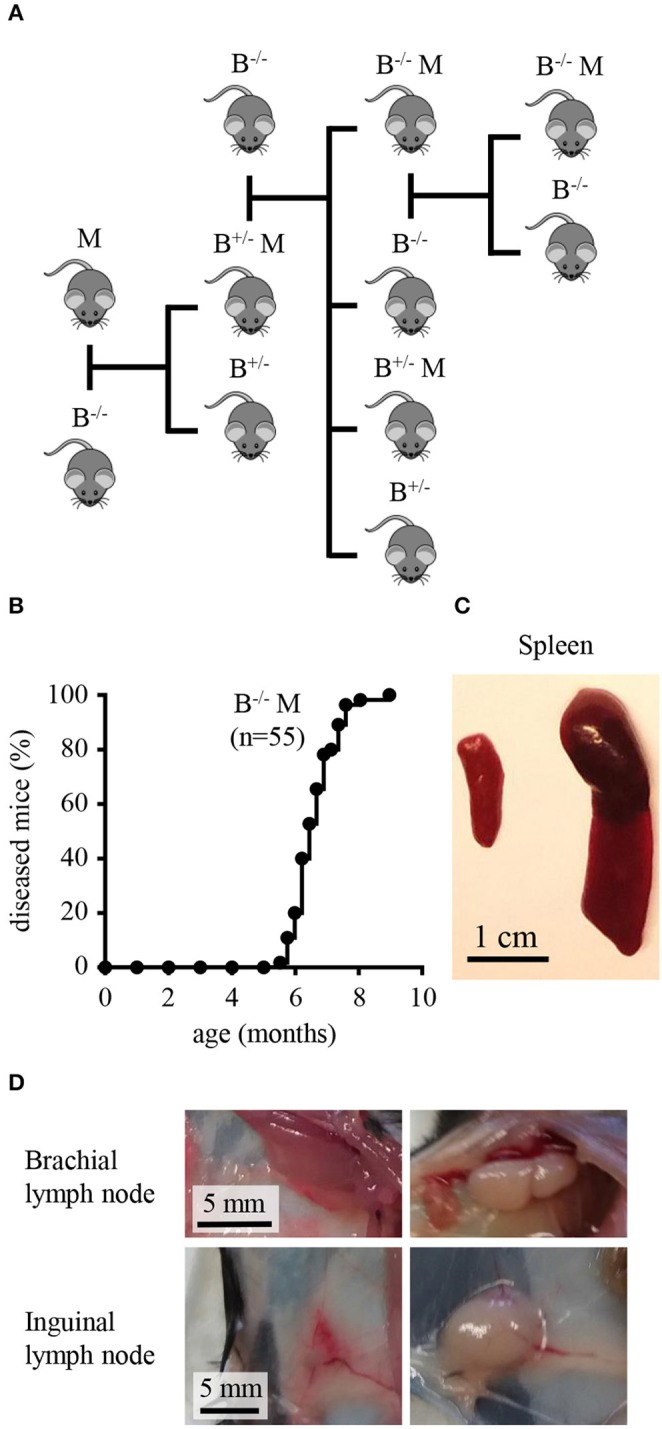
Batf3^−/−^ Mushi1 mice develop splenic tumors with occasional peripheral lymphadenopathy. **(A)** Breeding strategy to generate the new Batf3^−/−^ Mushi1 mouse strain. Mushi1 mice are heterozygous for a transgenic construct which contains the SV40 Large T-oncogene under the control of the murine CD11c promoter. Batf3^−/−^ mice (B^−/−^) were crossed with Mushi1 mice (M). Batf3^+/−^ Mushi1 mice (B^+/−^ M) from the offspring were then backcrossed with B^−/−^ mice. Starting from the second generation, the strain was maintained by breeding B^−/−^ M with B^−/−^ mice. **(B)** Age-dependent cumulative percentage of diseased Batf3^−/−^ Mushi1 mice. **(C,D)** Compared with healthy Batf3^−/−^ Mushi1 controls (left), diseased mice (right) show **(C)** spleen enlargement **(D)** and occasionally develop peripheral lymphadenopathy.

In the Mushi1 mouse, the development of splenic tumors is invariably caused by spl-cDC1 transformation (42). In this study, we aimed to design a strategy to favor the transformation of spl-cDC2s over spl-cDC1s with the purpose of generating new spl-cDC2-like MutuDC lines. To do this, we crossed Mushi1 mice with Batf3^−/−^ mice (Figure 1A) to introduce the CD11c:SV40LgT transgenic construct in the Batf3^−/−^ genetic background where all the cDC1s are selectively absent (26, 46).

Batf3^−/−^ Muhi1 mice started showing signs of histiocytosis between 6 and 8 months of age (Figure 1B), with a delay of around 3 months if compared with Mushi1 mice (42). Similarly to sick Mushi1 mice, diseased Batf3^−/−^ Mushi1 mice developed splenic tumors characterized by splenomegaly (Figure 1C). Additionally, in Batf3^−/−^ Mushi1 mice the progression of the disease was occasionally associated with peripheral lymphadenopathy (Figure 1D) that instead was never observed in Muhi1 mice.

**Figure 2:**
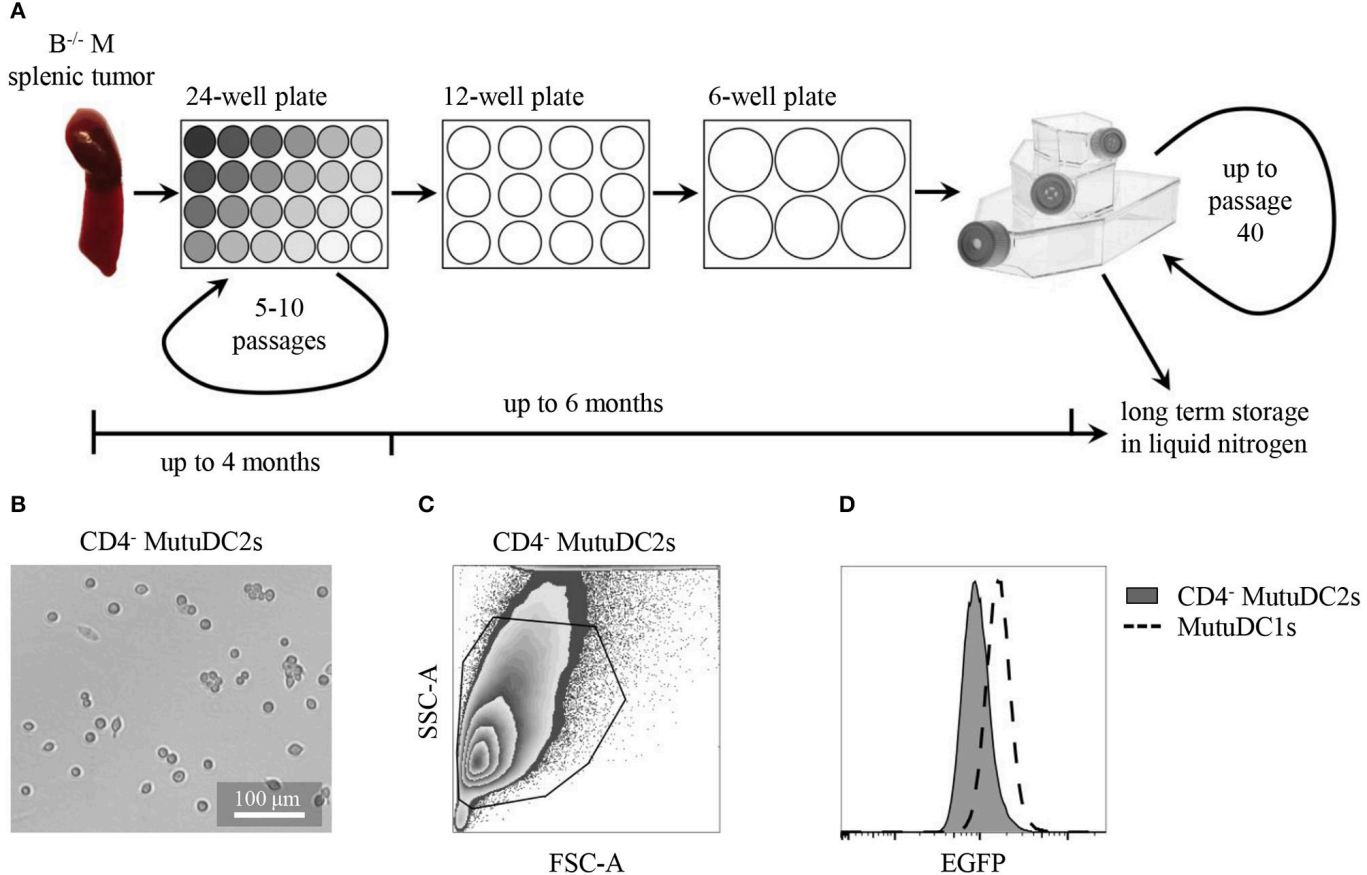
CD4^−^ MutuDC2s are derived from Batf3^−/−^ Mushi1 splenic tumors. **(A)** Schematic representation of CD4^−^ MutuDC2-derivation procedure. Splenocytes from Batf3^−/−^ Mushi1 (B^−/−^ M) splenic tumors were isolated and seeded in serial two-fold dilutions in 24-well plates. The adherent cells were maintained in 24-well plates for 5–10 passages until they became accustomed to culture conditions. The cultures were progressively expanded into plates and flasks with larger growth surfaces and frozen or used for experiments. **(B)** Light microscopy image of CD4^−^ MutuDC2s. **(C)** Flow cytometric analysis and gating of CD4^−^ MutuDC2s. **(D)** Flow cytometric comparison of expression of the SV40LgT-associated reporter EGFP in CD4^−^ MutuDC2s and in MutuDC1s.

Cell line derivation from Batf3^−/−^ Mushi1 splenic tumors was carried out as previously described (44, 45) and is schematized in Figure 2A. This procedure allowed to generate numerous immortalized cell lines from several mice. After a first exploratory phenotypic analysis, the most promising cell line, that in this work is named CD4^−^ MutuDC2 line, was chosen for further phenotypic and functional characterization. At the steady state, the stable CD4^−^ MutuDC2s have round morphology with few or absent dendritic processes (Figure 2B). They are adherent and have a slight tendency to cluster (Figure 2B). In conditions of prolonged culture, the CD4^−^ MutuDC2s can be split at densities as low as 1.5 × 10^4^ cells/cm^2^ and maintain their phenotype and function for at least 40 passages. With the increment of passage number, moderate increase of clustering tendency and slight reduction of adherence can be observed.

When analyzed by flow cytometry, the CD4^−^ MutuDC2s were found to be homogeneous (Figures 2C,D) and to express the SV40LgT-associated reporter EGFP even if at lower levels than the previously derived MutuDC1s (Figure 2D). This observation indicates lower expression of SV40LgT in the CD4^−^ MutuDC2s than in the MutuDC1s. In Mushi mice, higher levels of SV40LgT correspond to earlier onset of histiocytosis suggesting a dose-dependency of SV40LgT (42). Therefore, the lower expression of SV40LgT observed in the CD4^−^ MutuDC2s might explain the delayed development of splenic tumors in Batf3^−/−^ Mushi1 mice.

### CD4^−^ MutuDC2s are phenotypically similar to CD4^−^ spl-cDC2s

**Figure 3:**
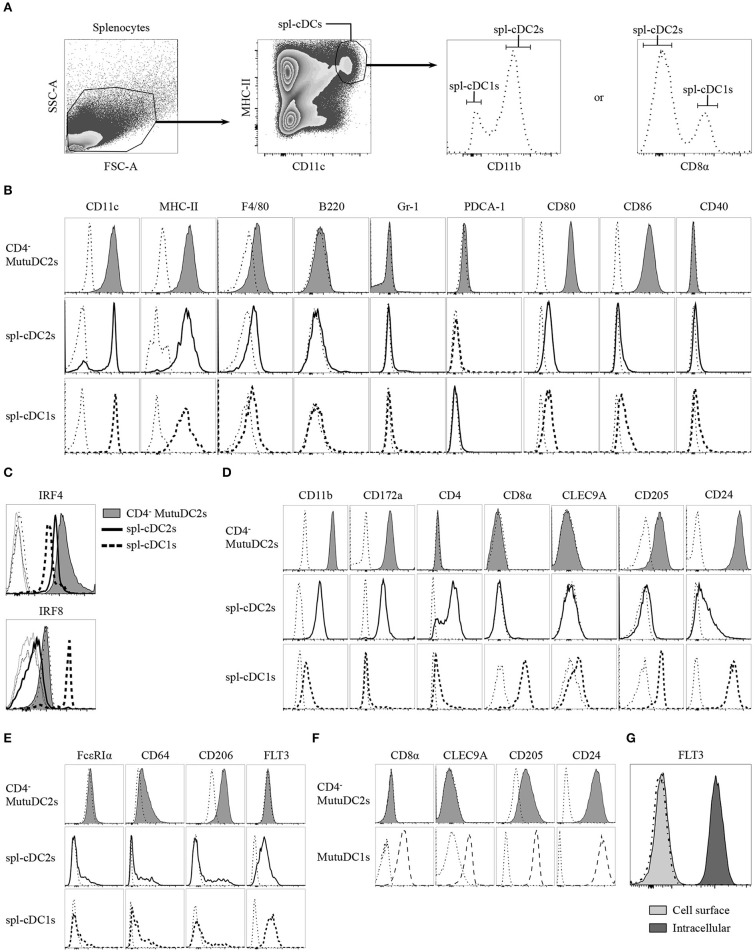
CD4^−^ MutuDC2s share the surface and intracellular marker expression profile with CD4^−^ spl-cDC2s. **(A–E)** Splenocytes from C57BL/6 mice were isolated by digestion of spleens with collagenase D followed by filtration through a 40 μm cell strainer. CD4^−^ MutuDC2s, MutuDC1s and splenocytes were stained with different antibody cocktails that always contained anti-MHC-II and anti-CD11c antibodies to distinguish spl-cDCs. Antibodies specific for either CD8α or CD11b were included in every staining cocktail to identify spl-cDC1s and spl-DC2s. The cells were analyzed by flow cytometry. **(A)** Gating strategy to discriminate spl-cDCs and their subsets. The dash-dotted lines show the fluorescence-minus-one (FMO) controls acquired for each marker. **(B)** CD4^−^ MutuDC2s and spl-cDC subsets were compared for the expression of the indicated surface markers. The gating strategies applied to analyze CD11c and MHC-II expression in the different spl-cDC subsets are detailed in Supplementary Figure 1. The dash-dotted lines show the FMO controls acquired for each marker. **(C)** CD4^−^ MutuDC2s and spl-cDCs were compared for the expression of the spl-cDC subset-specific transcription factors IRF4 and IRF8. FMO controls: CD4^−^ MutuDC2s (dash-dotted line), spl-DC1s (dotted line), spl-DC2s (thin solid line). **(D)** CD4^−^ MutuDC2s and spl-cDC subsets were compared for the expression of the indicated spl-cDC subset-specific surface markers. The dash-dotted lines show the FMO controls acquired for each marker. **(E)** CD4^−^ MutuDC2s and spl-cDC subsets were compared for the expression of the indicated moDC or spl-cDC characterizing surface markers. The dash-dotted lines show the FMO controls acquired for each marker. **(F)** CD4^−^ MutuDC2s and MutuDC1s were compared for the expression of the indicated spl-cDC1-specific surface markers. The dash-dotted lines show the FMO controls acquired for each marker. **(G)** Intracellular analysis of FLT3 expression in CD4^−^ MutuDCs. The dash-dotted line shows the background fluorescence of unstained cells. All the results are representative of two to six independent experiments.

To assess the phenotypic resemblance of the CD4^−^ MutuDC2s to spl-cDCs, we analyzed their expression of numerous extracellular and intracellular markers in comparison to fresh spl-cDCs. To do this, splenocytes from C57BL/6 mice were isolated, stained with fluorochrome-conjugated monoclonal antibodies and compared by flow cytometry with equally stained CD4^−^ MutuDC2s (Figures 3A–E). Spl-cDCs were defined as CD11c^hi^MHC-II^hi^ cells, and within this population, spl-cDC1s and spl-DC2s were distinguished on the basis of either CD8α or CD11b expression (Figure 3A). We observed that, similarly to fresh spl-cDCs, the CD4^−^ MutuDC2s are CD11c^hi^MHC-II^hi^F4/80^lo/−^B220^−^Gr-1^−^PDCA-1^−^ (Figure 3B). At the steady state, the expression of the co-stimulatory molecules CD80 and CD86 was found to be higher than in spl-cDCs in which the levels of these markers were low but detectable (Figure 3B). By contrast, CD40 expression, that was low in spl-cDCs, was not detectable in the CD4^−^ MutuDC2s (Figure 3B).

As mentioned, IRF8 and IRF4 have a primary role in the development of cDC1s and cDC2s, respectively (24). Fully differentiated cDC1s and cDC2s maintain a divergent expression of these two transcription factors. Indeed, spl-cDC1s express high levels of IRF8 and display low IRF4 expression, while, by contrast, both the CD4^+^ and the CD4^−^ spl-cDC2s have low levels of IRF8 and higher expression of IRF4 (31, 42), even though IRF4 deficiency affects more severely the development of the former subset (31). In order to correctly determine the belonging of the CD4^−^ MutuDC2s to either the spl-cDC1 or the spl-cDC2 subset, we analyzed them for the expression of IRF4 and IRF8. Our results showed that the CD4^−^ MutuDC2s express high levels of IRF4 and low levels of IRF8, with a profile that mirrors almost perfectly the pattern observed in spl-cDC2s as opposed to spl-cDC1s (Figure 3C). Consequently, we concluded that the CD4^−^ MutuDC2s belong to the spl-cDC2 subset.

To further prove the phenotypic similarity of the CD4^−^ MutuDC2s to spl-cDC2s we analyzed them for the expression of several spl-cDC subset-characterizing markers in comparison with fresh spl-cDC1s and spl-cDC2s. The spl-cDC2-specific markers CD11b and CD172a (47–50) appeared to be strongly expressed in the CD4^−^ MutuDC2s at an even higher level than in spl-cDC2s, while CD4 expression was found to be absent comparably to the CD4^−^ subpopulation of spl-cDC2 (Figure 3D). Analysis of the spl-cDC1-specific markers CD8α, CLEC9A, CD205, and CD24 (40, 51–53) revealed that the CD4^−^ MutuDC2s are CD8α^−^ and CLEC9A^−^ but express CD205 and CD24 at higher levels than the spl-cDC2s (Figure 3D and Supplementary Figure 2). However, consistently with a spl-cDC2 phenotype, the expression of these two markers is lower in the CD4^−^ MutuDC2s than in the MutuDC1s (Figure 3F).

Since some characteristics of the CD4^−^ MutuDC2s are consistent with the hypothesis of their belonging to the moDC subset, we analyzed them for the expression of moDC- or cDC-distinguishing markers to exclude the possibility of a monocytic origin. The two markers FcεRIα and CD64, which are considered very specific for moDCs (20), are respectively negative and low in the CD4^−^ MutuDC2s, with expression profiles that are very close to the ones observed in spl-cDCs (Figure 3E). CD206, known as macrophage mannose receptor (MMR), is also considered a moDC-characterizing marker even if its expression in cDC subsets is debated (20, 54–57). Analysis of CD206 expression in the CD4^−^ MutuDC2s showed that they express CD206 at low levels (Figure 3E) in line with a small cell population that is present within the cDC gate (Figures 3A,E). One of the main inducers of DC development is the the FMS-like tyrosine kinase 3 ligand (FLT3L) (58, 59). Therefore, its receptor FLT3 is one of the most characteristic markers of DCs. Accordingly, when analyzed by flow cytometry, spl-cDCs were found to be FLT3^+^ (Figure 3E). Surprisingly, the analysis of surface levels of FLT3 on the CD4^−^ MutuDC2s showed no detectable expression of this marker (Figures 3E,G). However, intracellular analysis of its expression in the CD4^−^ MutuDC2s revealed high intracellular levels of FLT3 (Figure 3G).

### CD4^−^ MutuDC2s are weakly activated by poly(I:C) or flagellin and express low levels of TLR3 but have high expression of TLR5

**Figure 4:**
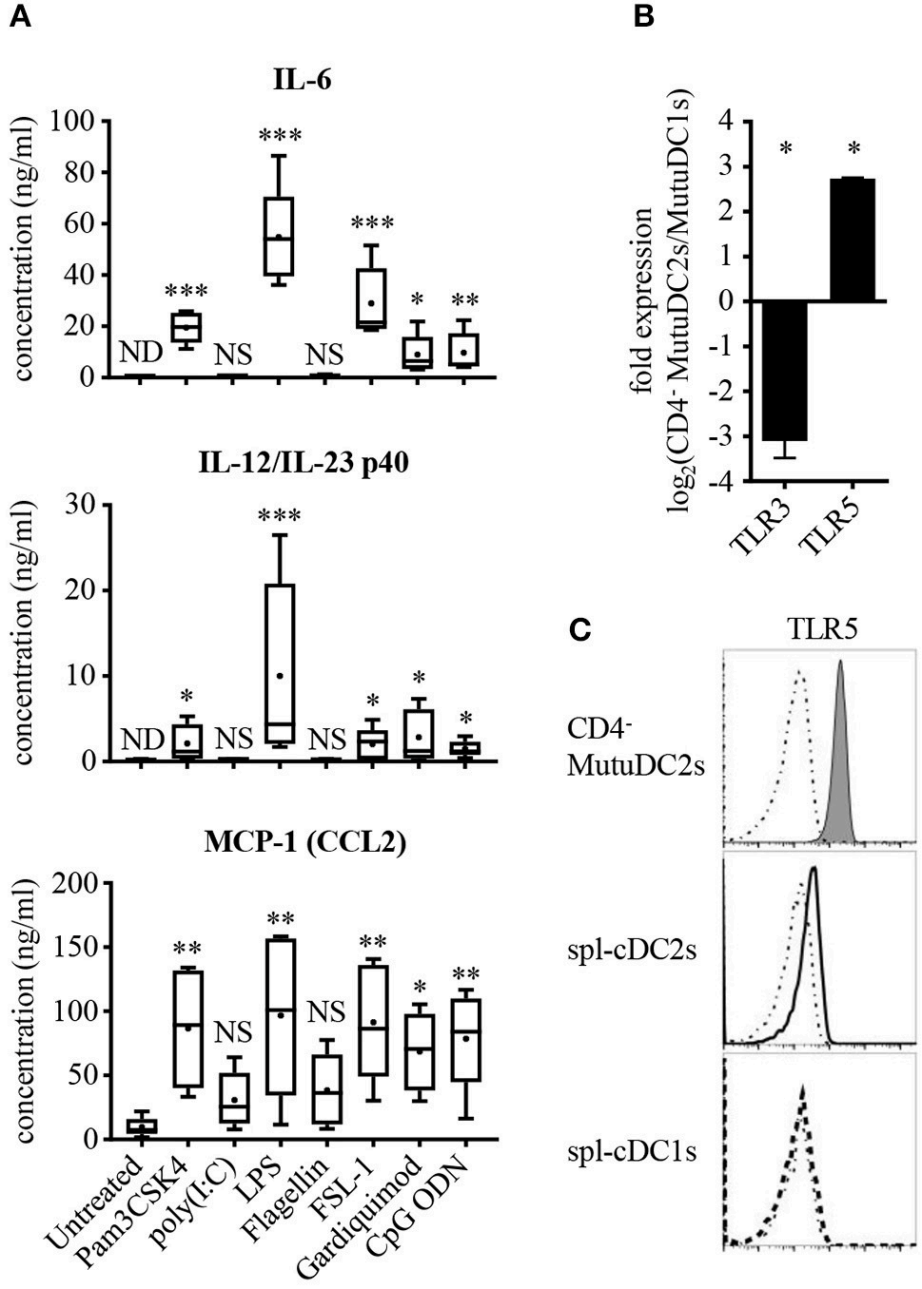
CD4^−^ MutuDC2s respond weakly to TLR3 and TLR5 ligands and have low TLR3 expression but high levels of TLR5. **(A)** CD4^−^ MutuDC2s were stimulated with specific TLR ligands: TLR1/2 ligand Pam3CSK4, TLR3 ligand poly(I:C), TLR4 ligand LPS, TLR5 ligand flagellin, TLR2/6 ligand FSL-1, TLR7 ligand Gardiquimod, TLR9 ligand CpG ODN. After 24 h, the supernatants were collected and analyzed by ELISA to determine the concentration of IL-6, IL-12/IL-23 p40, and MCP-1(CCL2). The graphs show the results of five independent experiments represented as box-and-whiskers plots: mean (+), median (horizontal line), interquartile range (box), min/max values (whiskers). To assess significance, every tested condition was compared with the untreated control by Kruskal-Wallis testing followed by uncorrected Dunn's testing (*p < 0.05, **p < 0.01, ***p < 0.001, NS, not significant; ND, not detectable). For the statistical analysis, all the measures below the lower detection limit of the assay were replaced with the value of the detection limit. **(B)** Expression of TLR3 and TLR5 was measured by RT-qPCR in CD4^−^ MutuDC2s compared with MutuDC1s. Fold expression in CD4^−^ MutuDC2s relative to MutuDC1s was calculated with the 2^−ΔΔ*Ct*^ method using β-actin expression as a reference for normalization. Data are presented as mean and SD of the log_2_ fold expression values from two independent experiments. The results were analyzed by two-tailed unpaired *t*-testing (*p < 0.05). **(C)** Flow cytometric analysis of TLR5 expression in CD4^−^ MutuDC2s compared with spl-cDC1s and spl-cDC2s. The isolation of splenocytes and the staining were carried out as described in Figure 3. The dash-dotted lines show the FMO controls not stained with anti-TLR5 antibody. The results are representative of two independent experiments.

The existence of several distinct DC subsets reflects a great variety of differential functional specificities, including fundamental aspects related to pathogen sensing and cytokine production (34, 35). Spl-cDC1s are known to be the only spl-DC subset to express TLR3 (36) and represent one of the primary producers of IL-12p70 during inflammation. By contrast, spl-cDC2s lack TLR3 expression and are weak producers of IL-12p70 (33, 36–38), but express IL-6, IL-23, and MCP-1(CCL2) (42, 60, 61). To assess the TLR expression profile of the CD4^−^ MutuDC2s and their ability to respond to TLR stimulation, we treated them with different TLR ligands and measured their production of IL-6, IL-12/IL-23 p40, IL-12p70, and MCP-1(CCL2) (Figure 4A and data not shown). Additionally, considering the ability of some DC subsets to produce the anti-inflammatory cytokine IL-10 in response to TLR stimulation (62), we measured also IL-10 levels in the supernatants of stimulated CD4^−^ MutuDC2s. The majority of the tested TLR ligands induced robust production of IL-6 and MCP-1(CCL2). With a similar ligand-dependent pattern, modest IL-12/IL-23 p40 secretion was observed, especially following treatment with the TLR4 ligand LPS to which the MutuDC1s respond weakly (44). However, after stimulation of the CD4^−^ MutuDC2s with the TLR3 ligand poly(I:C) or the TLR5 ligand flagellin, we measured only low or undetectable production of IL-6, IL-12/IL-23 p40, and MCP-1(CCL2) (Figure 4A). These findings indicated a limited capacity of the cells to respond to TLR3 and TLR5 stimulation. In addition, none of the tested TLR ligands induced production of IL-12p70 and IL-10 at detectable levels. By contrast, in the same conditions the MutuDC1s responded mainly to TLR3 and TLR9 ligands by producing IL-12/IL-23 p40, IL-12p70, IL-6, and IL-10, while they failed to secrete detectable levels of MCP-1(CCL2) (data not shown).

We reasoned that a low expression of TLR3 and TLR5 could explain the weak responsiveness of the CD4^−^ MutuDC2s to poly(I:C) and flagellin. To test this possibility, we measured by RT-qPCR the levels of TLR3 and TLR5 mRNAs in the CD4^−^ MutuDC2s in comparison to the MutuDC1s which are known to have high expression of TLR3 (44) and low levels of TLR5 (unpublished RNA-seq data-sets). In line with our hypothesis, TLR3 expression was found to be around eight-fold lower in the CD4^−^ MutuDC2s than in the MutuDC1s (Figure 4B). Surprisingly, the CD4^−^ MutuDC2s showed five- to six-fold higher expression of TLR5 than the MutuDC1s (Figure 4B). Flow cytometric comparison of the CD4^−^ MutuDC2s with fresh spl-cDCs further confirmed this observation by showing that TLR5 levels are higher in the CD4^−^ MutuDC2s than in spl-cDC2s (Figure 4C).

### CD4^−^ MutuDC2s are capable of MHC-I-Mediated and MHC-II-Mediated priming of CD8^+^ and CD4^+^ T cells but do not cross-present antigens through MHC-I

**Figure 5:**
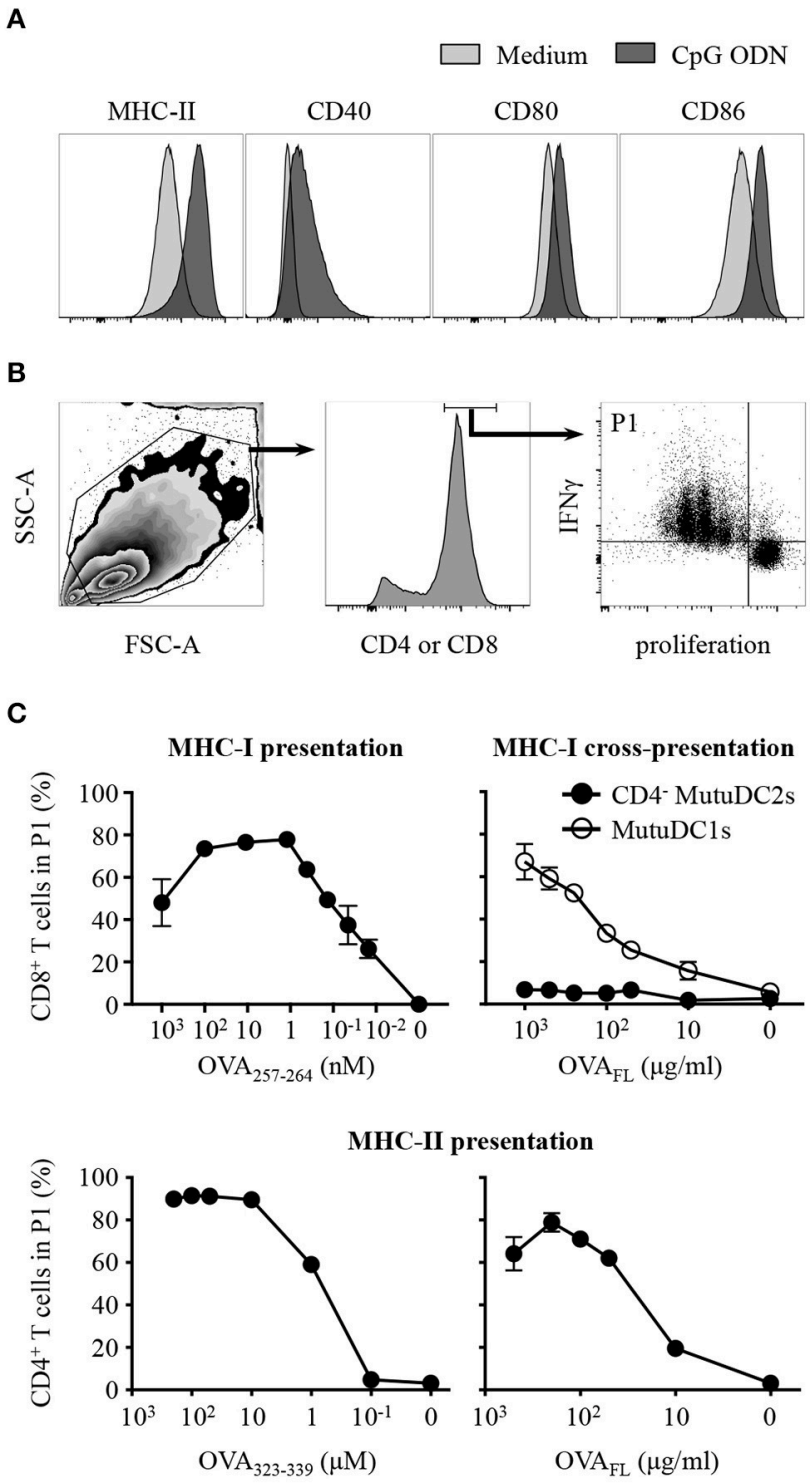
CD4^−^ MutuDC2s upregulate co-stimulatory molecules upon activation and display MHC-I-mediated and MHC-II-mediated activation of T cells but fail to cross-present antigens through MHC-I. **(A)** CD4^−^ MutuDC2s were incubated with medium or with CpG ODN. After 20 h they were stained with fluorescent-labeled antibodies specific for MHC-II, CD80, CD86, or CD40 and analyzed by flow cytometry. The graphs relative to untreated CD4^−^ MutuDC2s are the same shown in Figure 3B. **(B,C)** CD4^−^ MutuDC2s or MutuDC1s were pulsed, in the presence of CpG ODN, with full length ovalbumin (OVA_FL_) or with the MHC-I-restricted peptide OVA_257−264_ or with the MHC-II-restricted peptide OVA_323−339_ at the indicated concentrations. OVA-specific CD8^+^ or CD4^+^ T cells were isolated respectively from OT-I and OT-II mice and labeled with a proliferation dye. In the MHC-I T cell activation assay (**C**, top left), the CD8^+^ T cells were co-cultured for 3 days with OVA_257−264_-pulsed CD4^−^ MutuDC2s. In the MHC-I antigen cross-presentation assay (**C**, top right), the CD8^+^ T cells were co-cultured for 3 days with either OVA_FL_-pulsed CD4^−^ MutuDC2s or OVA_FL_-pulsed MutuDC1s. In the MHC-II T cell activation assay (**C**, bottom), the CD4^+^ T cells were co-cultured for 4 days with either OVA_323−339_-pulsed or OVA_FL_-pulsed CD4^−^ MutuDC2s. At the end of the co-cultures, the T cells were restimulated with PMA and ionomycin in the presence of brefeldin A and prepared for flow cytometric analysis by staining them with fluorescent-labeled antibodies specific for IFNγ and either CD4 or CD8α. **(B)** Gating strategy. The gate P1 contains the activated T cells defined as proliferating IFNγ^+^ cells. **(C)** The percentage of T cells in the gate P1 measured in each tested condition was plotted against the respective antigen concentration. The results are presented as mean and SD of technical triplicates and are representative of two to three independent experiments.

Upon activation, DCs undergo a process of maturation which entails several morphological and functional modifications which contribute to the efficient priming of T cells, including upregulation of co-stimulatory molecules, increase of surface MHC and development of dendrites (2, 63). To test the ability of our cell line to undergo such maturation, we activated the CD4^−^ MutuDC2s with CpG ODN and analyzed them by flow cytometry. The activated CD4^−^ MutuDC2s increased their adherence and showed modified morphology characterized by more tapered shape, enlarged size and presence of granules in the cytoplasm (data not shown). The levels of MHC-II, CD80 and CD86, which are already high in resting CD4^−^ MutuDC2s (Figure 3B), were increased after stimulation with CpG ODN (Figure 5A). Also CD40, which is not expressed by CD4^−^ MutuDC2s in the resting state (Figure 3B), was upregulated under these conditions (Figure 5A).

To test the ability of the CD4^−^ MutuDC2s to activate T cells, we pulsed them, in the presence of CpG ODN, with increasing concentrations of the ovalbumin (OVA)-derived peptides OVA_257−264_ or OVA_323−339_, which are respectively restricted to MHC-I and MHC-II. OVA-specific CD8^+^ or CD4^+^ T cells were isolated respectively from OT-I and OT-II mice, stained with a proliferation dye and co-cultured with OVA peptide-pulsed CD4^−^ MutuDC2s. The percentage of activated T cells, defined as proliferating IFNγ^+^ cells, was measured by flow cytometry (Figure 5B). This analysis showed that, in the presence of CD4^−^ MutuDC2s pulsed with increasing concentrations of MHC-I-restricted or MHC-II-restricted OVA peptides, the percentage of activated CD8^+^ or, respectively, CD4^+^ T cells increased accordingly (Figure 5C left, and Supplementary Figure 3). These results demonstrate that the CD4^−^ MutuDC2s have retained the ability to induce MHC-I-mediated and MHC-II-mediated T cell activation.

In an analogous experimental setup, we pulsed the CD4^−^ MutuDC2s with increasing concentrations of the full-length OVA (OVA_FL_). When co-cultured with OVA-specific CD4^+^ T cells, the OVA_FL_-pulsed CD4^−^ MutuDC2s induced T cell activation in an antigen concentration-dependent manner (Figure 5C bottom right and Supplementary Figure 3). Thus, this result not only confirmed that the CD4^−^ MutuDC2s have retained the capacity to activate T cells, but also showed that they are capable of endocytosing and processing extracellular antigens for presentation on MHC-II. By contrast, when we pulsed the CD4^−^ MutuDC2s with increasing concentrations of OVA_FL_ and co-cultured them with OVA-specific CD8^+^ T cells, we did not observe T cell activation. Instead, in the same conditions the MutuDC1s were able to induce CD8^+^ T cells to proliferate and produce IFNγ (Figure 5C top right and Supplementary Figure 3). These results showed that the CD4^−^ MutuDC2s are not capable of antigen cross-presentation in contrast to the MutuDC1s which are known to share this functional feature with their splenic counterpart (44).

## Discussion

DCs are well known for their scarcity *in vivo* that limits tremendously their accessibility for experimentation (64, 65). In addition, isolated DCs are very sensitive to long term culture and under these conditions they easily undergo functional modification, spontaneous activation and cell death (66, 67). For these reasons, significant efforts have been made to create suitable and reliable models for the study of DC biology. Several procedures have been proposed to simplify the access to sufficient amounts of viable and non-activated DCs for experimentation by differentiating DC progenitors *in vitro* and by expanding the number of DCs *in vivo* (68). However, despite their indisputable value, these methods still display disadvantages including the need for additional steps of cell isolation and purification, as well as the sensitivity, the functional instability and the limited lifespan in long term culture of the cells that they generate. Therefore, a widely pursued alternative to the *in vitro*-differentiation and *in vivo*-expansion strategies is represented by stable DC lines. Several approaches have allowed to generate stable DC lines (68) which in most cases have demonstrated to be very good models to study single aspects of DC biology (69–74). However, specific functional features of different DC subsets are often found concomitantly in several DC lines, raising doubts about their ability to maintain and fully represent the DC-subset functional specificities observed *in vivo* (75–83). Moreover, the need for special culture conditions, like growth at low permissive temperature (77, 83) or constant supplementation with growth factors (78, 84, 85), possibly represents an additional technical complication for the maintenance of some DC lines in long-term culture. Additionally, a relevant and yet often neglected caution is required when the capacity of the DC lines to maintain fully unaltered their phenotypic and functional properties over the passages, and especially at high passage number, has not been extensively ascertained.

Among the different examples of oncogene-based approaches to immortalize DC lines, several strategies are based on *ex vivo* transduction or transfection of DCs with the SV40LgT (77, 83, 86). Our approach is different because it is based on the generation of SV40LgT-transgenic mice and on the transgene-induced transformation of DCs *in vivo* with consequent development of DC tumors (42). Our group generated several SV40LgT-transgenic murine lines where the transgene expression was restricted to DCs (42). Among them we selected the one, the Mushi1 line, in which the transgene expression was sufficient to induce transformation of DCs and development of DC tumors, but not high enough to cause functional modifications of the cells. Finally, from the DC tumors we were able to derive the stable immortalized MutuDC lines (44). In parallel, we generated with a similar strategy several KO and transgenic MutuDC lines by crossbreeding the parental KO or transgenic murine lines with Mushi1 mice (44, 45). This crossbreeding approach can be applied to introduce virtually any kind of mutation in the cell lines or to derive them from any different genetic background. Thus, this strategy confers great versatility to our method and represents one of its main advantages. Additionally, the recent development and diffusion of genome-editing methods has enormously increased the possibilities to modify our cell lines *in vitro* (44, 45).

All the MutuDC lines generated so far from Mushi1 mice derive from spl-cDC1s (44), and therefore they are named MutuDC1s. To obtain new MutuDC lines with phenotype and functional properties of spl-cDC2s, we applied the crossbreeding strategy to Batf3^−/−^ mice which are devoid of spl-cDC1s (26, 46). As expected, the Batf3^−/−^ Mushi1 mice developed splenic tumors, even if with a delayed onset of the disease if compared with Mushi1 mice (42). Given the concentration-dependency of the SV40LgT (42), this difference can be explained by our previous observation that in Mushi1 mice the levels of the transgene expression are lower in spl-cDC2s than in spl-cDC1s (42). Consequently, the oncogenic transformation is likely to occur earlier in spl-cDC1s than in spl-cDC2s, possibly explaining why all the MutuDCs generated from Mushi1 mice derive from spl-cDC1s.

From the splenic tumors of Batf3^−/−^ Mushi1 mice, we derived the new CD4^−^ MutuDC2 line whose characterization is described in this work. The expression of the SV40LgT was measured in the CD4^−^ MutuDC2s through the analysis of its associated EGFP reporter. We found that the CD4^−^ MutuDC2s express lower levels of the transgene than the previously derived MutuDC1s, reflecting the difference observed between spl-cDC2s and spl-cDC1s in Mushi1 mice.

To assess the belonging of our CD4^−^ MutuDC2s to one of the spl-DC subsets, we analyzed their surface and intracellular marker expression profile and compared them with freshly isolated spl-DCs. The CD4^−^ MutuDC2s display high levels of CD11c and MHC-II with lack of the pDC-specific markers B220, Gr-1 and PDCA-1. Additionally, in their resting state they are CD80^hi^ and CD86^hi^. These observations demonstrated that the CD4^−^ MutuDC2s have a relatively mature spl-cDC phenotype. Analysis of expression of the subset-determining transcription factors IRF4 and IRF8 showed that, comparably to spl-cDC2s, the CD4^−^ MutuDC2s express high IRF4 and low IRF8. Therefore, we concluded that they belong to the spl-cDC2 subset. Additional surface marker analysis further corroborated this conclusion by showing that the CD4^−^ MutuDC2s strongly express the spl-cDC2-distinctive markers CD11b and CD172a and have low levels of CD4, comparably to the CD4^−^ spl-cDC2 subset. The markers CD8α, CLEC9A, CD205, and CD24 are known to be specifically expressed by spl-cDC1s (40, 51–53). In our CD4^−^ MutuDC2s, CD8α and CLEC9A are not expressed, but the levels of CD205 and CD24 are higher than in fresh CD4^−^ spl-cDC2s. However, the comparison between the CD4^−^ MutuDC2s and the MutuDC1s shows that the expression of CD205 and CD24 is still lower in the former, in agreement with the differences observed *in vivo* between spl-cDC2s and spl-cDC1s. It should be mentioned that the levels of CD205 are also higher in the MutuDC1s if compared with freshly isolated spl-cDC1s (44). This increase of CD205 expression both in the CD4^−^ MutuDC2s and in the MutuDC1s reminds of what is observed in overnight cultures of *ex vivo* spl-cDCs in the presence of GM-CSF, where CD205 expression is upregulated both in spl-cDC2s and in spl-cDC1s but remains higher in the latter (87). Concerning CD24 expression in the CD4^−^ MutuDC2s, we cannot fully explain the upregulation of this marker. However, it has been shown that CD24^int^ and, to a lesser extent, CD24^hi^ DC precursors maintain the potential to generate spl-cDC2s (18). Moreover, several examples of CD11b^+^CD24^+^ cDC2s, which share at least partially developmental origin, phenotype and functional characteristics with spl-cDC2s (29, 23, 32, 35), can be found among the mucosal cDC populations in lung (29, 88), small intestine (32), and nose (89).

Many of the phenotypic characteristics that we described are consistent with a moDC phenotype. However, in Mushi1 mice, SV40LgT expression is driven by the 5.7 kb CD11c proximal promoter which restricts the transgene expression to DCs (43). Indeed, the SV40LgT-associated reporter, the IRES-linked EGFP, is not detectable in CD11c^lo^ macrophages and monocytes, demonstrating no expression of the transgene in these cell types (unpublished results). This makes it highly unlikely that the cell line here described is monocyte-derived. Consistently, the analysis of the moDC-specific markers Ly-6C (Gr-1), FcεRIα, and CD64 (20), which are negative or low in the CD4^−^ MutuDCs, highlights this conclusion. In further agreement with the results discussed above, the CD4^−^ MutuDCs express high levels of the DC-defining marker FLT3, in spite of an unusual intracellular localization. When we generate DC lines, they become able to grow at low densities only after several passages. We think that this is due to the fact that, during the derivation process, we select for cells that can secrete growth factors that favor DC growth. Consistently, at low densities the cells preferentially grow in conditioned medium. A likely growth factor is FLT3L whose binding to its receptor FLT3 has been shown to induce the dimerization and internalization of FLT3 (90). Therefore, the presence of FLT3L in the culture medium would explain the intracellular localization of FLT3 that we observe in the CD4^−^ MutuDC2s.

Distinct profiles of TLR and cytokine expression represent an additional characterizing difference between splenic DC subsets. For instance, spl-cDC1s are known to express mainly TLR3 and TLR9 (36) and to be major producers of IL-12p70 (37, 38). On the contrary, they do not produce the chemokine MCP-1(CCL2) (42). The picture is reversed in spl-cDC2s which express a broader array of TLRs, with the well described exception of TLR3 (36), and produce MCP-1(CCL2), IL-6, and IL-23 but only limitedly IL-12p70 (37, 38, 60, 61). Consistently, after stimulation with the ligands of TLR1/2, TLR4, TLR2/6, TLR7, and TLR9, our CD4^−^ MutuDC2s responded by producing IL-6, MCP-1(CCL2), and IL-12/IL-23 p40 but did not express detectable levels of IL-12p70. Additionally, in line with the limited cytokine production observed after stimulation with the TLR3 ligand poly(I:C), transcription analysis showed very low levels of TLR3 mRNA. We also observed that the CD4^−^ MutuDC2s exhibit a generally weak responsiveness to treatment with the TLR5 ligand flagellin, in apparent contrast with their relatively high expression of TLR5.

Several studies in the past have analyzed TLR5 expression in different DC subsets. For example, intestinal lamina propria CD11b^+^ cDC2s show high levels of TLR5 and rely considerably on this receptor for detection of pathogens, maturation and induction of cytokine production (70, 91, 92). By contrast, our knowledge is less precise regarding spl-cDCs. Indeed, while some studies have shown that TLR5 is expressed by spl-cDCs, in particular by spl-cDC2s (36, 93), in other cases TLR5 was found to be very low or absent (70, 91, 92, 94). Interestingly, just one study could show *in vitro* a connection between TLR5 expression and direct flagellin-induced maturation of spl-cDCs (93), while in other reports this effect was not observed (94, 95). Additionally, even in the former case, flagellin always induced a very limited or absent production of cytokines like IL-12 and IL-6. These observations are consistent with a differential tissue- and DC subset-specific role of TLR5. In agreement with this hypothesis, TLR5 was shown to function also as an endocytic receptor that mediates the uptake of flagellin promoting MHC-II presentation of flagellin epitopes to CD4^+^ T cells (96). Further investigation of this model showed that the CD4^−^ spl-cDC2s are the main subset among the spl-cDCs to carry out this TLR5-mediated flagellin processing pathway (97). Our results show very low, and yet detectable, levels of TLR5 in spl-cDC2s as opposed to spl-cDC1s that are TLR5^−^ (Figure 4C). Instead, TLR5 expression is high in the CD4^−^ MutuDC2 line. However, the upregulation of TLR5 displayed by the CD4^−^ MutuDC2s, together with their low responsiveness to flagellin, appears to integrate perfectly in the context of the functional specialization of the CD4^−^ spl-cDC2s, rather than representing a divergence of the CD4^−^ MutuDC2 line from its splenic counterpart.

In addition to the induction of cytokine production, the encounter of DCs with a pathogen initiates a process of maturation that causes several functional modifications including upregulation of the co-stimulatory molecules CD40, CD80, and CD86, increase of the surface levels of MHC molecules and reduction of the antigen-uptake capacity of DCs, accompanied by an increase of their ability to process antigenic peptides and load them into MHC complexes for presentation to T cells (2, 7, 8, 9). All these elements are essential to ensure the efficient priming of T cells and to initiate an appropriate adaptive response. Upon treatment with CpG ODN, the CD4^−^ MutuDC2s upregulate CD40, CD80, and CD86 and increase their surface levels of MHC-II. When pulsed, in the presence of CpG ODN, with an ovalbumin-derived MHC-II-restricted peptide or with the full-length ovalbumin, they are able to take up the antigen and present it to antigen-specific CD4^+^ T cells. We speculate that the need of a rather high antigen concentration in our presentation assays could be linked to the relatively mature phenotype that the CD4^−^ MutuDC2s show at the steady state, which might imply a reduced endocytic capacity in favor of a higher antigen processing and presentation efficiency. The CD4^−^ MutuDC2s also proved to be very efficient in the direct MHC-I-mediated activation of CD8^+^ T cells. Therefore, their inability to cross-present antigens to CD8^+^ T cells, even at the highest concentrations, mirrors precisely the functional distinction that is observed *in vivo* between the cross-presenting spl-cDC1s and the non-cross-presenting spl-cDC2s and further distinguishes the CD4^−^ MutuDC2s from moDCs (19, 56, 98).

In conclusion, we have exploited the versatility of our SV40LgT-based derivation method to generate the new CD4^−^ MutuDC2 line which has striking phenotypic and functional resemblance to the CD4^−^ spl-cDC2 subset. This cell line has already proven to be a reliable model for the study of cDC2s. Indeed, in a recent report from our group, resistance to collagen induced arthritis (CIA) was partially recovered by the adoptive transfer of CD4^−^ MutuDC2s in a CIA-susceptible CD11b^−/−^ mouse model, highlighting a potential tolerogenic role of cDC2s in the modulation of autoimmune responses (99). Furthermore, the CD4^−^ MutuDC2s have been successfully transduced with a CRISPR-Cas9 editing system to generate a new CD11b^−/−^ CD4^−^ MutuDC2 line (unpublished data). As we have shown, the new CD4^−^ MutuDC2s are simple to culture and can be expanded at will, providing a virtually unlimited source of cells for experimentation. They can be easily manipulated with standard experimental techniques and display great stability of their phenotypic and functional properties in long term culture as long as their passage number is maintained below 40-50. For these reasons the CD4^−^ MutuDC2s represent a new valuable tool for the study of DC biology.

## Data availability statement

The raw data supporting the conclusions of this manuscript will be made available by the authors, without undue reservation, to any qualified researcher.

## Author contributions

MP designed and performed experiments and data analysis, wrote the article and revised the reviewed drafts. DA generated the Batf3^−/−^ Muhi1 mouse line and participated in the cell line derivation. MS helped with the experimental design and performed preliminary experiments. HA-O conceived the project, acquired the founding, supervised experimental design and data analysis and reviewed the article drafts. All the authors reviewed the submitted version of the article.

### Conflict of interest statement

The authors declare that the research was conducted in the absence of any commercial or financial relationships that could be construed as a potential conflict of interest.
