# Supplementary material for: Establishment and Characterization of a Functionally Competent Type 2 Conventional Dendritic Cell Line
Source: Front Immunol. 2018 Aug 24;9:1912. doi: 10.3389/fimmu.2018.01912 (PMC6117413; doi:10.3389/fimmu.2018.01912)
Supplement: Supplementary file 1 [file Data_Sheet_1.PDF]

Supplementary Material

Establishment and Characterization of a Functionally Competent Type 2 Conventional Dendritic Cell Line

Matteo Pigni, Devika Ashok, Mathias Stevanin, Hans Acha-Orbea*

* **Correspondence:** Hans Acha-Orbea: Hans.Acha-Orbea@unil.ch

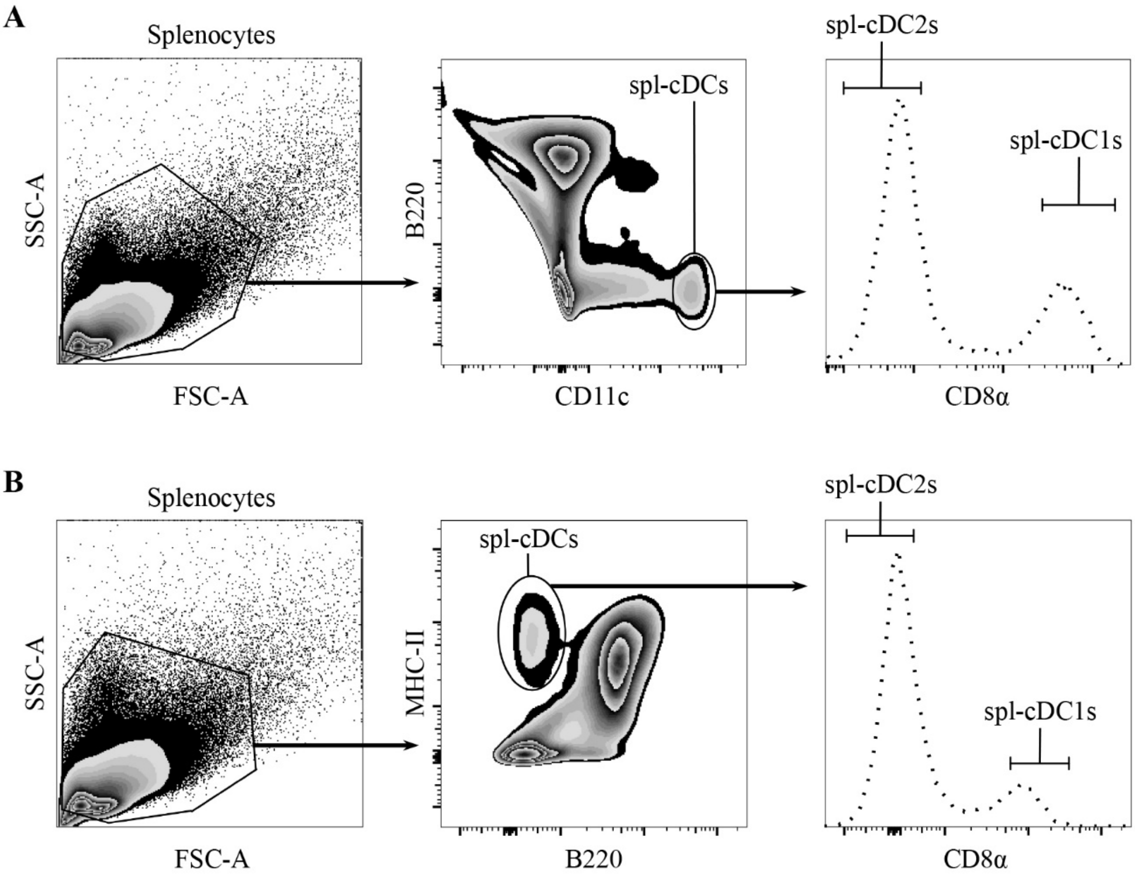

Supplementary Figure 1. Gating strategy for the analysis of CD11c and MHC-II expression in spl-cDC subsets. Splenocytes from C57BL/6 mice were isolated by digestion of spleens with collagenase D followed by filtration through a 40 μm cell strainer. The cells were analyzed by flow cytometry after staining with fluorescent-conjugated antibodies specific for B220, MHC-II, CD11c and CD8α. Spl-cDCs were identified through the analysis of either (**A**) B220 and CD11c expression or (**B**) MHC-II and B220 expression. (**A,B**) In both cases, the spl-cDC subsets were distinguished on the basis of CD8α expression.

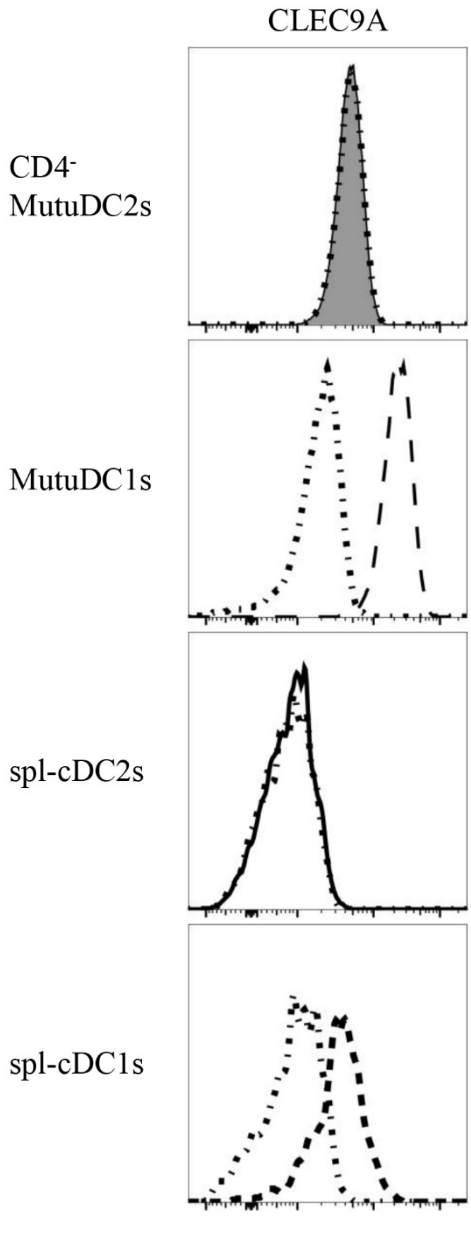

Supplementary Figure 2. CD4^{-} MutuDC2s do not express CLEC9A. Splenocytes were isolated as described in **Figure S1**. CD4^{-} MutuDC2s, MutuDC1s and splenocytes were analyzed by flow cytometry after staining with fluorescent-conjugated antibodies specific for MHC-II, CD11c, CD8α and CLEC9A. The spl-cDC subsets were identified through the analysis of MHC-II, CD11c and CD8α expression as reported in **Figure 3**. The dash-dotted lines show the fluorescence-minus-one controls not stained with anti-CLEC9A antibody. The results are representative of two independent experiments.

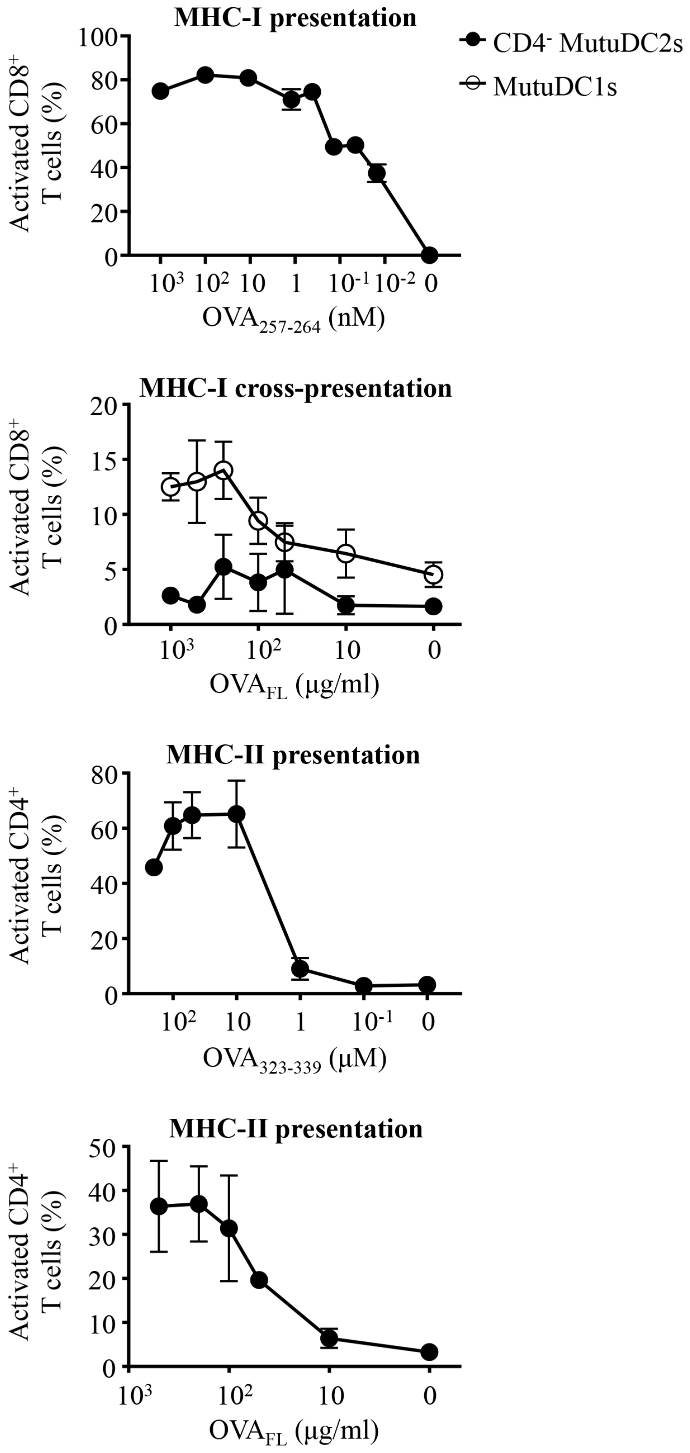

Supplementary Figure 3. CD4$^-$ MutuDC2s activate T cells through MHC-I and MHC-II but do not cross-present peptides through MHC-I. Biological replicate of the experiment illustrated in **Figure 5C**. The results are presented as mean and SD of technical triplicates and are representative of two to three independent experiments.
